# Supplementary material for: The interactome of intact mitochondria by cross-linking mass spectrometry provides evidence for coexisting respiratory supercomplexes
Source: Mol Cell Proteomics. 2017 Dec 8;17(2):216–32. doi: 10.1074/mcp.RA117.000470 (PMC5795388; doi:10.1074/mcp.RA117.000470)
Supplement: Supplemental Data [file supp_RA117.000470_133922_0_supp_23978_fzffwf.zip › spectra_annotation/mito_DR_spectra_annotation/65-1-4-1-3-1.pdf]

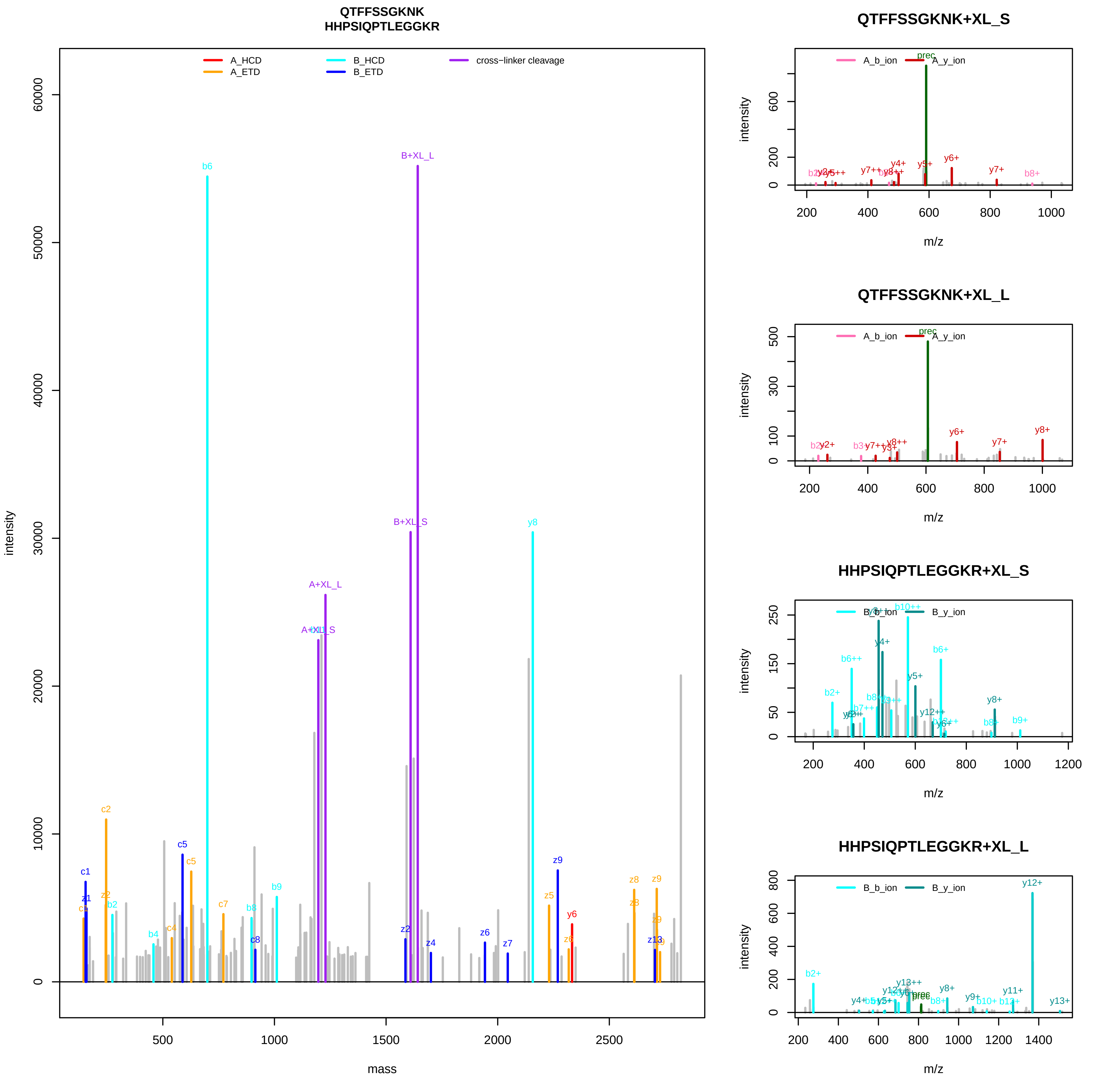
QTFFSSGKNK
HHPSIQPTLEGGKR
A_HCD
A_ETD
B_HCD
B_ETD
cross−linker cleavage
intensity
mass
0
10000
20000
30000
40000
50000
60000
500
1000
1500
2000
2500
b6
B+XL_L
B+XL_S
y8
A+XL_L
A+XL_S
c2
c5
z9
c1
z1
c5
b9
z8
z9
z5
y6
c7
b8
b2
b4
c4
c8
z2
z4
z6
z7
z13
QTFFSSGKNK+XL_S
QTFFSSGKNK+XL_L
HHPSIQPTLEGGKR+XL_S
HHPSIQPTLEGGKR+XL_L
A_b_ion
A_y_ion
B_b_ion
B_y_ion
prec
m/z
y6+
y7+
y8+
y4+
y5+
b8+
b2+
b6++
b6+
b10++
y5+
y8+
b9+
y12+
y13++
b8+
y9+
y11+
y13+
b10+
b12+
